# Fatty acid microemulsion for the treatment of neonatal conjunctivitis: quantification, characterisation and evaluation of antimicrobial activity

**DOI:** 10.1007/s13346-016-0338-3

**Published:** 2016-10-20

**Authors:** Ummara Butt, Amr ElShaer, Lori A. S. Snyder, Athina Chaidemenou, Raid G. Alany

**Affiliations:** 1 Drug Discovery, Delivery and Patient Care (DDDPC) Theme, School of Pharmacy and Chemistry, Kingston University London, Penrhyn Road, Kingston upon Thames, Surrey KT1 2EE UK; 2 School of Life Sciences, Kingston University, Kingston upon Thames, KT1 2EE UK; 3 School of Pharmacy, University of Auckland, Auckland, New Zealand

**Keywords:** Gas chromatography, GC-FID, Fatty acids, Fatty acid methyl esters (FAME), FAME preparation, Method development, Design of experiment (DoE), Validation characteristics

## Abstract

Fatty acids (FAs) are used by many organisms as defence mechanism against virulent bacteria. The high safety profile and broad spectrum of activity make them potential alternatives to currently used topical antibiotics for the treatment of eye infections in neonates. The current study utilised a Design of Experiment approach to optimise the quantification of five fatty acids namely; lauric acid, tridecanoic acid, myristoleic acid, palmitoleic acid and α-linolenic acid. The significance of the influence of the experimental parameters such as volume of catalyst, volume of n-hexane, incubation temperature, incubation time and the number of extraction steps on derivatisation was established by statistical screening with a factorial approach. Derivatisation was confirmed using attenuated total reflectance infrared (ATR) and 1H NMR spectrum. A gas chromatographic method (GC-FID) was developed and validated according to ICH guidelines for the identification and quantification of fatty acids. The results were found to be linear over the concentration range studied with coefficient of variation greater than 0.99 and high recovery values and low intra-day and inter-day variation values for all FAs. Then, different α-linolenic acid-based microemulsions (MEs) were prepared using Tween 80 as surfactant, polyethylene glycol 400 (PEG 400) as co surfactant and water as aqueous phase. The developed GC method was used to quantify the FA content in ME formulations. The results indicated that the developed GC method is very effective to quantify the FA content in the ME formulations. The antimicrobial efficacy of FA-based MEs were tested against *Staphylococcus aureus*. It was concluded that the FA-based MEs have strong antimicrobial effect against *S. aureus*.

**Electronic supplementary material:**

The online version of this article (doi:10.1007/s13346-016-0338-3) contains supplementary material, which is available to authorized users.

## Introduction

Eye infections in newborns (28 days or younger) are recognised as a severe form of conjunctivitis (neonatal conjunctivitis or so called ophthalmia neonatorum). Symptoms include conjunctival hyperaemia, inflammation eye lid swelling and yellowish purulent discharge. Infants become infected when born to a mother with a sexually transmitted disease caused by chlamydia, herpes, streptococcus and *Staphylococcus aureus*. The first line of treatment for neonatal conjunctivitis of bacterial origin involves the use of silver nitrate solution or topical antibiotics such as 1 % tetracycline or 0.5 % erythromycin eye ointments [1]. However, the use of broad spectrum ophthalmic ointments is in continuous decline mainly because of emergence of bacterial resistance. An ocular prophylaxis or treatment of neonatal conjunctivitis based on fatty acids and their derivatives offers a viable alternative to conventional antibiotics-based treatment regimens. Fatty acids are widely occurring in natural fats and dietary oils and play an important role as nutritious substances and metabolites in living organisms. Many fatty acids are considered to have antibacterial and antifungal properties as well. In recent years, microbicidal effects of medium- and long-chain fatty acids and their corresponding 1-monoglycerides have been studied. They have been found to have a broad spectrum of microbicidal activity against enveloped viruses and various Gram positive and Gram negative bacteria in vitro, including pathogens such as *Neisseria gonorrhoeae*, *Candida albicans* and *S. aureus* [2–4]. Because of the potential of FAs in treating eye infections and the possibility of formulating them as topical ocular dosage forms, it is important to develop a precise, accurate and robust analytical method to quantify them.

There are several analytical methods commonly used to quantify fatty acids, including gas chromatography with flame ionization detector (GC-FID), gas chromatography coupled with mass spectrometry (GC-MS), high-performance liquid chromatography (HPLC), nuclear magnetic resonance (NMR) spectroscopy, silver ion high-performance liquid chromatography and silver ion thin-layer chromatography [5]. Amongst all of these analytical methods, GC-MS and GC-FID are the most popular techniques for analysing essential oils and food for fatty acid content [6–8]. GC provides excellent separation and quantification together with good sensitivity and also offers convenience and relatively low cost compared to other methods [9, 10]. GC-FID is a conventional method to separate and determine fatty acids in biological samples [11–13]. Although a gas chromatography/mass spectrometry (GC-MS) method has been developed to quantitatively determine medium and long chain (C_8_–C_26_) fatty acids [14], the GC analysis with flame ionization detection (GC-FID) is still the most frequently used method because of its high sensitivity and accuracy [15]. Fatty acids can be analysed as free fatty acids (underivatised) or as fatty acid methyl esters (derivatised) by gas chromatography [16]. However, fatty acids in their underivatised form have higher polarity and tend to form hydrogen bonds that lead to absorption issues, thus making them difficult to analyse [17]. The polar carboxyl functional groups must first be neutralised to distinguish between the very slight differences exhibited by unsaturated fatty acids; so, derivatisation in GC is commonly undertaken to increase the volatility or decrease the polarity of the analytes of interest for resolved peaks with better separation. Therefore, derivatisation is usually required to make fatty acids more suitable for qualitative and/or quantitative analysis [18].

A method for preparing methyl esters of fatty acids should be simple, fast and quantitative and should not give rise to structural changes and unwanted side reactions [19]. Fatty acid methyl esters (FAMEs) are normally prepared by acid-catalysed methanolysis or alkali-catalysed methanolysis. Base-catalysed methanolysis uses sodium hydroxide (NaOH) or potassium hydroxide (KOH) in methanol. Acid-catalysed methanolysis commonly uses methanolic hydrogen chloride, methanolic sulfuric acid or boron trifluoride (BF_3_) in methanol [20–24]. Boron trifluoride (BF_3_) in methanol is a commonly used acidic catalyst for the esterification of fatty acids for GC analysis, but the production of methoxy-substituted fatty acids as artefacts was reported during the esterification of unsaturated fatty acids with BF_3_-methanol [25, 26]. Boron trichloride (BCl_3_) in methanol is also used for the esterification of fatty acids for GC analysis [27]. Out of these methods, BCl_3_ in methanol was found to be the most efficient catalyst for the esterification of fatty acids for GC analysis because of the speed with which the esterification is affected [28, 29]. Moreover, it was reported that the formation of the methoxy artefacts with BCl_3_-methanol is much less compared to BF_3_-methanol [30].

Microemulsions are pharmaceutical preparations consisting of oil, water, surfactant and co-surfactant. Microemulsions offer several advantages over other formulations such as enhanced drug solubility, good thermodynamic stability, ease of preparation, elegant appearance and increased permeation across the cornea [31, 32].

The current study reports a simple derivatisation method using BCl_3_-methanol for accurate and reliable identification and quantification of a mixture of fatty acids by GC-FID. In this study, fatty acid derivatisation was optimised using a quality by design approach. The influence of the experimental parameters such as volume of catalyst, volume of n-hexane, incubation temperature, incubation time and the number of extraction steps was evaluated by statistical screening with a factorial approach. Different FA-based microemulsions (MEs) were prepared. FA was used as an oil phase, Tween 80 as surfactant and polyethylene glycol 400 (PEG 400) as co-surfactant with water as aqueous phase. Finally, antibacterial activity of the FA-based MEs was tested against *S. aureus*.

## Materials and methods

### Materials

Lauric acid (LA) 12:0, tridecanoic acid (TA) 13:0, myristoleic acid (MOA) 14:1, palmitoleic acid (POA) 16:1, α-linolenic acid (ALA) 18:3 and internal standard (IS) pentadecanoic acid (15:0) were purchased from Sigma (Sigma Aldrich, UK); all standards were of purity ≥99 % (GC). The derivatisation reagent, BCl_3_-methanol, 12 % *w*/*w* (12 % boron trichloride in methanol), n-hexane (HPLC grade, purity, ≥99 %), Tween 80, PEG 400 and anhydrous sodium sulphate were also purchased from Sigma (Sigma-Aldrich, UK).

### Organism

Fresh cultures of *S. aureus* strain (NCTC06571) were grown on nutrient agar (Oxoid CM 0003, Oxoid Basingstoke, UK) plates at 37 °C for 24 h. The colonies were removed from the culture plate with a loop and suspended into a 3-ml Ringer solution until cloudy. The culture was mixed well, and the standard density was adjusted to 0.5 McFarland (1.5 × 10^8^ CFU per ml).

### Methods

#### Derivatisation and GC analysis of FAs

##### Preparation of fatty acid methyl esters (FAMEs)

A stock solution of the five fatty acids at 1 mg ml^−1^ concentration was prepared by solubilising 10 mg of each of the five fatty acids in 10 ml n-hexane. Working standard solutions were prepared by diluting a standard stock solution in n-hexane (100 μg ml^−1^) and were subjected to derivatisation conditions. In short, fatty acid solutions were treated with various volumes (0.5–2 ml) of BCl_3_-methanol (12 % *w*/*w*). Then, samples were heated to 50–60 °C for 5–8 min. Then, samples were extracted with n-hexane (1–2 ml) and water by hand-shaking for 1 min until both layers were clear. The layers were allowed to settle, and upper (organic) layer was transferred into a clean vial. Then, the organic layer containing FAMEs was dried by adding 500 mg anhydrous sodium sulphate.

##### Gas chromatography (GC) analysis of FAMEs

The FAME analysis was performed on a gas chromatography system (Shimadzu GC-2014, Japan) equipped with an auto sampler injector (AOC-20i) and a flame ionization detector (SFID1). Substances were separated on a capillary column (BPX5, 30 m × 0.25 mm i.d., film thickness 0.25 μm). The injection volume was 0.5 μl, which was used with a split ratio of 1:50. The injection port was heated at 280 °C and a flame ionization detector operated at 280 °C. The oven temperature programming used was 180 °C, then increased to 280 °C at 20 °C/min and held for 5 min. The total run time was 5 min. Helium at a flow rate of 1.5 ml min^−1^ was used as carrier gas. Fatty acids were identified and quantified on the basis of their retention times.

##### Nuclear magnetic resonance (NMR) spectroscopy

To confirm the formation of FAMEs, the methyl esters derived from fatty acid samples were additionally analysed by attenuated total reflectance infrared (ATR) and nuclear magnetic resonance (NMR) spectroscopy. ^1^H NMR analyses were performed using a Bruker (Billerica, MA, USA) ARX-400 spectrometer under ambient conditions. One to two milligrams of the fatty acids and fatty acid methyl esters were dissolved in 1 ml of deuterated solvent and placed in sample capillary vial up to 5–10-cm height. Deuterated chloroform (CDCl_3_) was used as solvent which also served as internal reference (shift value of residual proton at 7.27 ppm).

##### Attenuated total reflectance infrared (ATR) spectroscopy

Samples were prepared by solubilising 10 mg of each of the five fatty acids in 1 ml of ethanol. Then, a drop of the sample was placed on a transparent glass disc and allowed to air-dry. ATR spectra were recorded on Thermo Scientific Nicolet iS5 FT-IR Spectrometer (Madison, WI USA) in the range 4000–400 cm^−1^. EZ OMNNIC 7.0 software was used to interpret the IR spectrum.

### Design of experiment and data analysis

The optimisation of derivatisation method was carried out through design of experiment (DoE) to analyse the effect of different parameters on the derivatisation of fatty acid sample. The experimental design and data analysis were performed using statistical software package “Minitab 17” (Minitab Inc., UK) [33]. A two-level five-factor fractional factorial design (2^5–2^) was applied in this experiment. In the present study, the FAME derivatisation was optimised by looking at the effect of five independent variables, namely volume of catalyst (X_1_), volume of n-hexane (X_2_), reaction temperature (X_3_), reaction time (X_4_) and the number of extraction steps (X_5_) on one dependent response, peak area of the analysed FAMEs using GC-FID. A total of eight experiments were performed prior to optimisation based on a two-level five-factor fractional factorial design (2^5–2^). The five variables were taken at two levels, low and high, which were represented by transformation values of −1 and +1, respectively, as shown in Table 1. Eight samples were prepared according to the procedure described above. In order to minimise the effect of unexplained variability in the response because of external factors, the experiments were randomised and obtained in triplicate. During the optimisation of the derivatisation, GC peaks were identified on the basis of their retention times. Table 2 shows the eight combinations of the different levels of the five variables investigated.

**Table 1:** Independent variables, their actual and coded values

Coded value	Independent variables				
	Volume of catalyst (X_1_) ml	Volume of n-hexane (X_2_) ml	Reaction temp (X_3_) °C	Reaction time (X_4_) min	Extraction steps (X_5_)
−1	0.5	1	50	5	2
1	2	2	60	8	4

**Table 2:** Experimental design showing the various independent variables used in the optimisation of derivatisation method

Sample number	X_1_	X_2_	X_3_	X_4_	X_5_
1	1	1	1	1	1
2	1	−1	1	−1	1
3	1	−1	−1	−1	−1
4	1	1	−1	1	−1
5	−1	−1	1	1	−1
6	−1	1	−1	−1	1
7	−1	1	1	−1	−1
8	−1	−1	−1	1	1

### Validation procedure

The optimal derivatisation conditions were applied to validate the GC-FID method. GC-FID method was fully validated according to the International Conference on Harmonisation (ICH) guidelines. A standard mixture containing five FAs at concentration of 1 mg ml^−1^ was prepared. Five calibration standard solutions (200 to 25 μg ml^−1^) were prepared from stock solution by diluting with n-hexane, and FAMEs were prepared under optimised conditions. The analysis was carried out using GC under the same conditions described above. Calibration curves were constructed by plotting the relative responses for each analyte versus its concentration. The validation parameters such as response linearity, sensitivity, limit of detection (LOD) and quantification (LOQ), recovery and precision of the analytical procedure were calculated.

### Microemulsion preparation and characterisation

#### Construction of pseudo-ternary phase diagram

A pseudo-ternary phase diagram was constructed to determine the concentration range of all components (α-linolenic acid (ALA)/surfactant/co-surfactant/water) in which they form a microemulsion. The pseudo-ternary phase diagram was constructed by using the phase diagram by micro-plate dilution (PDMPD) method, a novel technique based on the water titration method [34]. The surfactant and co-surfactant were mixed at a 1:1 ratio. Different mixtures of FA and surfactant/co-surfactant mixtures were prepared at weight ratios of 0.5:9.5, 1:9, 2:8, 3:7, 4:6, 5:5, 6:4, 7:3, 8:2 and 9:1. The microtitre plates were filled by a pipette in accordance with the filling scheme: the FA–Tween 80/PEG 400 (S/CoS) mixture was added starting at A1 with 200 μl up to B8 with 10 μl, decreasing 10 μl in each well, and then water was added from A2 with 10 μl up to B9 with 200 μl, increasing 10 μl in each well. The wells C1 up to D8 were filled with the next batch using the same procedure. After the plates had been filled, they were sealed and then shaken for 24 h at room temperature (25 °C). After that, the microplates were characterised by measuring absorbance using microplate reader and by making a visual evaluation of the isotropy and the border between the homogeneous or the heterogeneous system.

### Preparation of microemulsion formulations

According to microemulsion region in the phase diagram, three ME formulations were selected at different component ratios. Linolenic acid was used as an oil phase, Tween 80 as surfactant and PEG 400 as co-surfactant with water as aqueous phase. The composition of three ME formulations is given in Table 4. Linolenic acid was dissolved under stirring in mixture of Tween 80 and PEG 400. Then, the appropriate amount of water was added to the mixture drop by drop with continuous stirring.

### Characterisation of microemulsions

#### Droplet size and zeta potential measurement

The droplet size, polydispersity index (PDI) and zeta potential (ZP) of MEs were measured by dynamic light scattering using a Zetasizer (Malvern instruments Ltd., Malvern, UK). ME samples were analysed in triplicate at 25 °C.

### Drug content determination

Concentration and drug content of FA-based ME formulations were determined using the developed GC method. ME samples were derivatised with BCl_3_-methanol (12 % *w*/*w*) under optimised conditions. Then, samples were extracted with n-hexane (1–2 ml) by hand-shaking for 1 min until both layers were clear. The layers were allowed to settle, and the upper (organic) layer was transferred into a clean vial. The organic layer containing FAMEs was dried by adding 500 mg of anhydrous sodium sulphate. Then, samples were analysed by the developed GC method as described above.

### Antibacterial activity of FAs and microemulsions against *S. aureus*

The antimicrobial activity of five FAs (lauric acid, tridecanoic acid, myristoleic acid, palmitoleic acid, α-Linolenic acid), microemulsions and its individual components against *S. aureus* were checked using disc diffusion method. FA solutions (1 and 5 mM) were prepared by solubilising an appropriate amount of each FA in 100 % ethanol. Blank paper discs (6 mm diameter) were loaded with 10 μl of the prepared stock solutions of FAs and microemulsion formulations and allowed to air-dry at room temperature. Nutrient agar plates were inoculated with bacterial suspension by dipping a sterile cotton-wool swab into the suspension and spreading the inoculum evenly over the entire surface of the plates by swabbing in three directions. Plates were allowed to dry before applying discs. Then, the discs containing the test agents were applied to the surfaces of inoculated plates. Plates were inverted and incubated at 37 °C for 24 h to allow for bacterial growth. Inhibition zone diameters (IZD) were measured in millimetres.

## Results and discussion

### Investigating the influence of various factors on fatty acid derivatisation

Various parameters such as catalyst volume, solvent volume, reaction temperature, reaction time and number of extraction steps were investigated to select the optimum conditions in order to obtain a high degree of accuracy, sensitivity and reliability for the determination and quantification of the five fatty acids. The results of the partial factorial design are summarised in Supplementary Table 1 and expressed as peak areas of the five fatty acids. The coefficient values and probability of significance (*P* value) are also shown in Supplementary Table 2.

### Effect of reaction temperature and time on fatty acid derivatisation

Reaction temperature and time are important aspects in esterification of fatty acids and are closely related to each other. In order to determine optimum reaction temperature and time, the samples were heated at different temperatures (50–60 °C) for different time intervals (5–8 min). The results showed that an increase in the reaction temperature and reaction time results in an increase in the degree of esterification for most of the fatty acids and this was reflected on the peak area of the analysed samples. Nonetheless, the effect of both the reaction temperature and incubation time was insignificant (*P* > 0.05) for all the analysed samples (Supplementary Table 2).

Several studies in the literature have reported that the temperature causes significant changes in the fatty acid composition in cooking oil. Moreover, high temperature can alter the geometry and position of double bonds in fatty acids [35, 36]. It was reported that the formation of additional conjugated linolenic acid isomers and artefacts was lower at 40 °C than methylation at 60 and 80 °C though the base and acid-catalysed methylation at 40 °C resulted in a lower derivatisation yield [37]. The study concluded that increasing the reaction temperature is associated with an increase in the degree of esterification of the most of the fatty acids. On the other hand, increasing the reaction time was associated with an increase in the peak area for short chain fatty acids: lauric acid C12:0 and tridecanoic acid C13:0. This effect was reverted upon increasing the chain of the fatty acids and increasing the degree of unsaturation. On contrary to the previous study, our results did not show any significant effect of esterification temperature or reaction time on the yield of derivatisation [37]. Nonetheless, the selected conditions evaded any artefact formations as confirmed by the ATR and NMR data.

### Effect of volume of catalyst (BCl_3_-methanol) on fatty acid derivatisation

Many studies evaluated the effect of catalyst type on the esterification of fatty acids; for instance, the study conducted by Araujo et al. assessed the effect of boron trihalide type on methylation of fatty acids, and the study concluded that the two halides used BF_3_ and BCl_3_ have no significant effect [38]. Nevertheless, the study has not investigated the effect of the volume of the catalyst used. Besides, the unsaturated fatty acids reported to produce methoxy artefacts by addition of methanol across the double bond in the presence of high concentrations of acidic catalysts [27]. The volume of catalyst may significantly affect the derivatisation results. In order to understand how the volume of BCl_3_-methanol will affect the degree of derivatisation and artefact formation, catalyst volumes between 0.5–2 ml were studied (Supplementary Table 2). These results indicate that peak area of all five fatty acids is not affected by volume of catalyst used (*P* > 0.05) and has no effect on the effective methylation area as suggested by Araujo et al. [38].

### Effect of volume of solvent (n-hexane) on fatty acid derivatisation

In the current study, the effect of n-hexane volumes, 1 and 2 ml on the peak areas of the formed FAMEs, was investigated. The results showed that the peak area of four fatty acids is significantly affected (*P* value <0.05 for C12:0, C13:0, C14:1 and C18:3) by the volume of n-hexane (Supplementary Table 2). In contrast, C16:0 showed no significant changes in peak area (*P* > 0.05) by changing the volume of n-hexane. n-hexane was reported as the solvent of choice for extraction of fatty acids [38]. Table 3 shows that increasing the volume of n-hexane was associated with a dramatic drop in the formation of the FAMEs. Probably, increasing n-hexane volume is associated with decreasing the effective methylation area which in turn decreases the formation of FAMEs which is soluble in the n-hexane layer. The results come in line with Araujo et al. findings [38].

**Table 3:** Equations, correlation coefficients, intra-day and inter-day variation and recovery percentage for five fatty acids

Fatty acids	Calibration curve equation	Regression coefficient (*R* ^2^)	LOD	LOQ	(*n* = 6) Intra-day, RSD%^a^	(*n* = 6 + 6 + 6) Inter-day, RSD% ^b^	Accuracy R% ^c^
C12:0	y = 68,166x + 666.51	0.9938	0.015	0.046	2.49	3.44	97.98
C13:0	y = 69,869x + 458.17	0.9959	0.012	0.038	2.66	4.71	97.45
C14:1	y = 78,575x + 533.73	0.9966	0.011	0.034	4.31	3.02	98.64
C16:1	y = 88,538x + 96.649	0.9989	0.0064	0.019	2.88	3.57	100.38
C18:3	y = 77,175x + 827.61	0.9983	0.008	0.024	5.11	3.73	98.42

^a^The mean value of RSD established from six (*n* = 6) complete analyses of each sample in a day

^b^The mean value of RSD established from six complete analyses repeated three consecutive days

^c^The mean of recovery percentage established from the complete analysis in triplicate of FAME standard fortified with a standard working solution at three concentrations

### Effect of number of extraction steps on fatty acid derivatisation

Another important factor that may significantly affect the derivatisation results is the number of extraction steps. For this reason, it was decided to check whether two step or four step extraction process would improve the efficiency of derivatisation. The results showed (Supplementary Table 2) that peak area of four fatty acids C12:0, C13:0, C14:1 and C18:3 is significantly affected (*P* < 0.05) by changing the number of extraction steps whereas C16:0 shows no significant changes in peak area (*p* > 0.05) by changing the number of extraction steps. Coefficient values demonstrated that the peak area of fatty acids is negatively correlated to the number of extraction steps. This suggests that as the number of extraction steps decrease, the FAME concentrations increase and vice versa.

All the aforementioned variables did not show any significant effect on the extraction of palmitoleic acid (C16:1). This could be attributed to the lower solubility of palmitoleic acid in n-hexane, and this could possibly be due to its higher melting point compared to other unsaturated fatty acids including myristoleic acid and α-linolenic acid. The fatty acid’s structure has an influence on its melting point. The behaviour of fatty acids depends on their unique hydrocarbon tails. Fatty acids with long, unsaturated tails tend to be less soluble and have higher melting points than those with shorter, saturated tails. However, the presence of cis double bonds in unsaturated fatty acids is associated with an increased solubility and decreased melting points of unsaturated fatty acids [39]. Moreover, the solubility of fatty acids decreases with carbon chain length and increases with the degree of unsaturation (number of double bonds) [40]. Both palmitoleic acid C16:1 (cis-Δ9) and myristoleic acid C14:1 (cis-Δ9) contain one double bond in their tail that occurs between carbons 9 and 10, but the carbon chain length of palmitoleic acid is higher than that of myristoleic acid so this could be the reason for lower solubility of palmitoleic acid in n-hexane as compared to myristoleic acid, whereas higher solubility of α-linolenic acid C18:3(cis-Δ9, cis-Δ12, cis-Δ15) in n-hexane could be due to the presence of three double bonds in its tail (degree of unsaturation).

### Optimization of fatty acid derivatisation

**Fig. 1:**
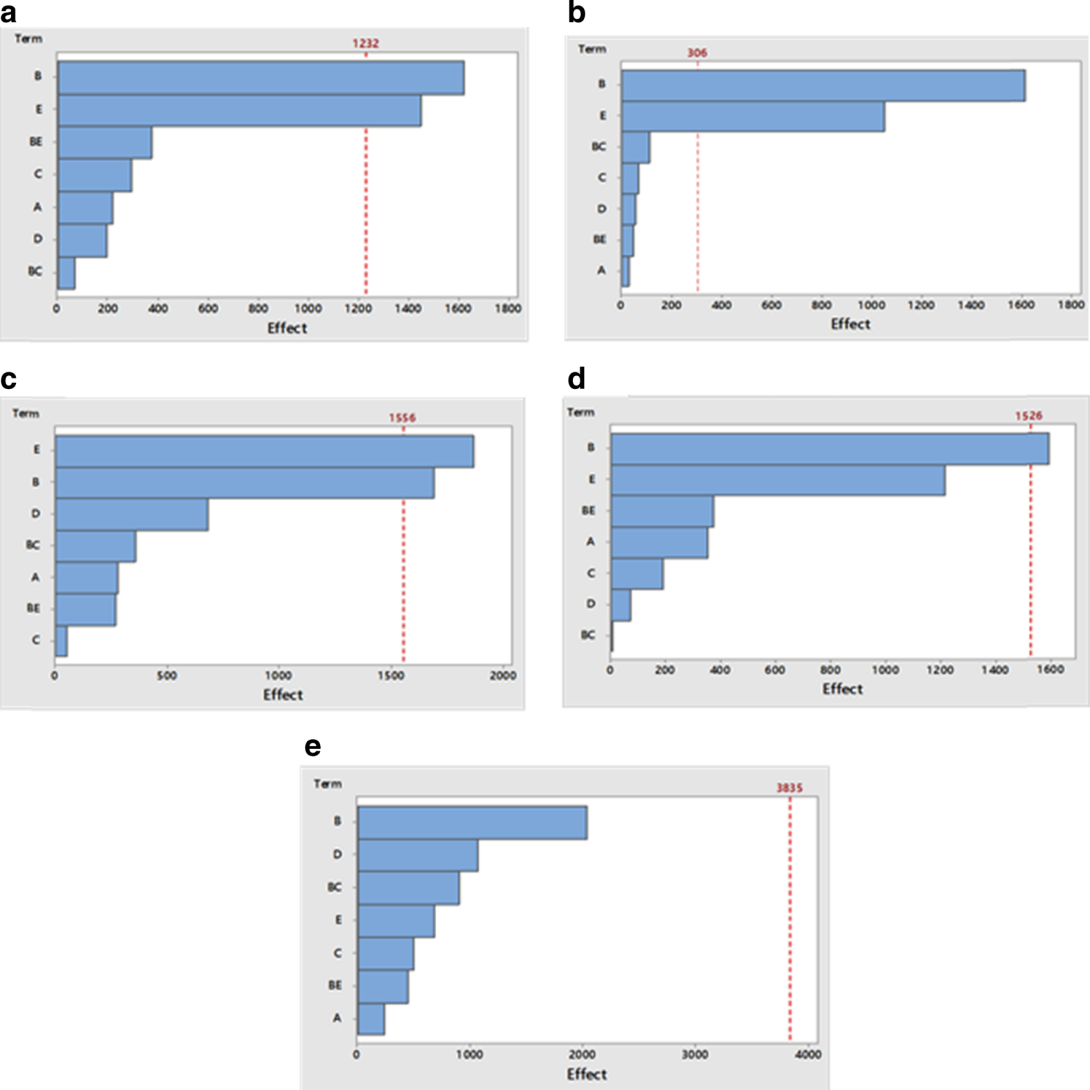
Pareto chart showing the effect of variables on the peak area of C12:0 (lauric acid; **a**), C14:1 (myrisoleic acid; **b**), C18:3 (α-linolenic acid; **c**), C13:0 (tridecanoic acid; **d**), C16:1 (palmitoleic acid; **e**) where volume of catalyst X1 (**a**), volume of n-hexane X2 (**b**), reaction temperature X3 (**c**), reaction time X4 (**d**) and the number of extraction steps X5 (**e**)

Pareto charts (Fig. 1) also confirmed that the peak area of four fatty acids, namely C12:0, C13:0, C14:1 and C18:3, was significantly affected by both factors B and E volume of n-hexane and number of extraction steps, respectively, as they have passed the red line and are considered as significant factors. Although C13:0 shows only slight changes (*P* = 0.043) in the peak area whereas the Pareto chart for C16:0 shows that peak area is not affected by any of these factors as none of the factors are extending beyond the reference line. It is noted that none of the two-way interactions are significant indicating that the effect of the process variable on the peak area is independent of the level of the others.

**Fig. 2:**
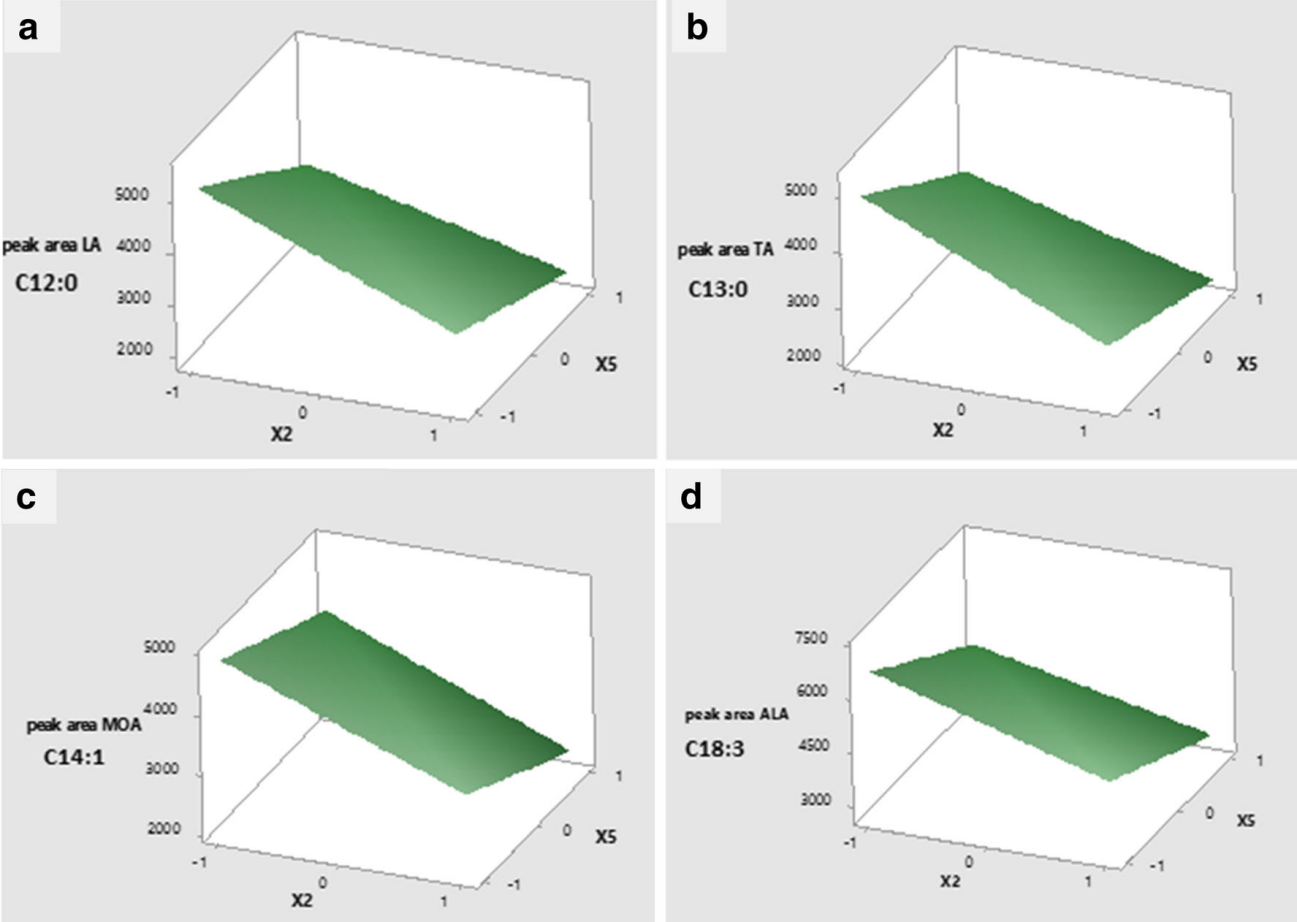
3D surface plots of significant (*P* < 0.05) interaction effects of volume of n-hexane and number of extraction step time on the peak area of C12:0, C13:0, C14:1 and C18:3

**Fig. 3:**
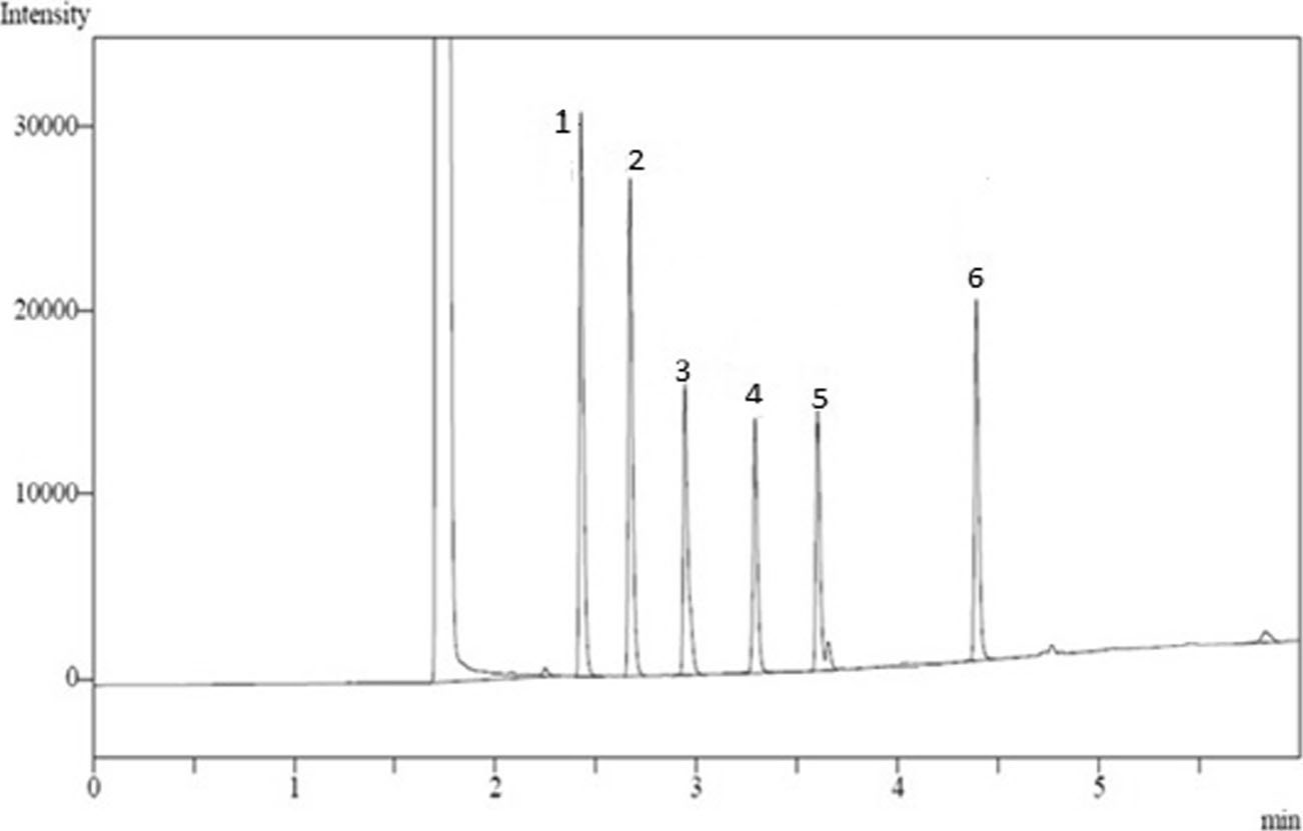
GC-FID chromatogram of fatty acids [peak before 1 = hexane, peak 1 = lauric acid (C12:0), peak 2 = tridecanoic acid (C13:0), peak 3 = myristoleic acid (C14:1), peak 4 = palmitoleic acid (C16:1), peak 5 = pentadecanoic acid (C15:0), peak 6 = α-linolenic acid (C18:3)]

Optimisation was also performed using 3D surface plots as shown in Figs. 2 and 3. These plots show how the volume of n-hexane and number of extraction steps affected the peak area of fatty acids. The 3D surface plots showed that the volume of n-hexane and the number of extraction steps have negative effect on the peak area of fatty acids where peak area increased as the volume of n-hexane and number of extraction steps decreased. This means that the fatty acid samples should be extracted in two steps rather than four steps (the peak area was higher when samples were extracted in 2 steps rather than 4 steps) with 1 ml rather than 2-ml volume of n-hexane (the peak area is higher at 1 ml than it is at 2-ml volume of n-hexane).

**Fig. 4:**
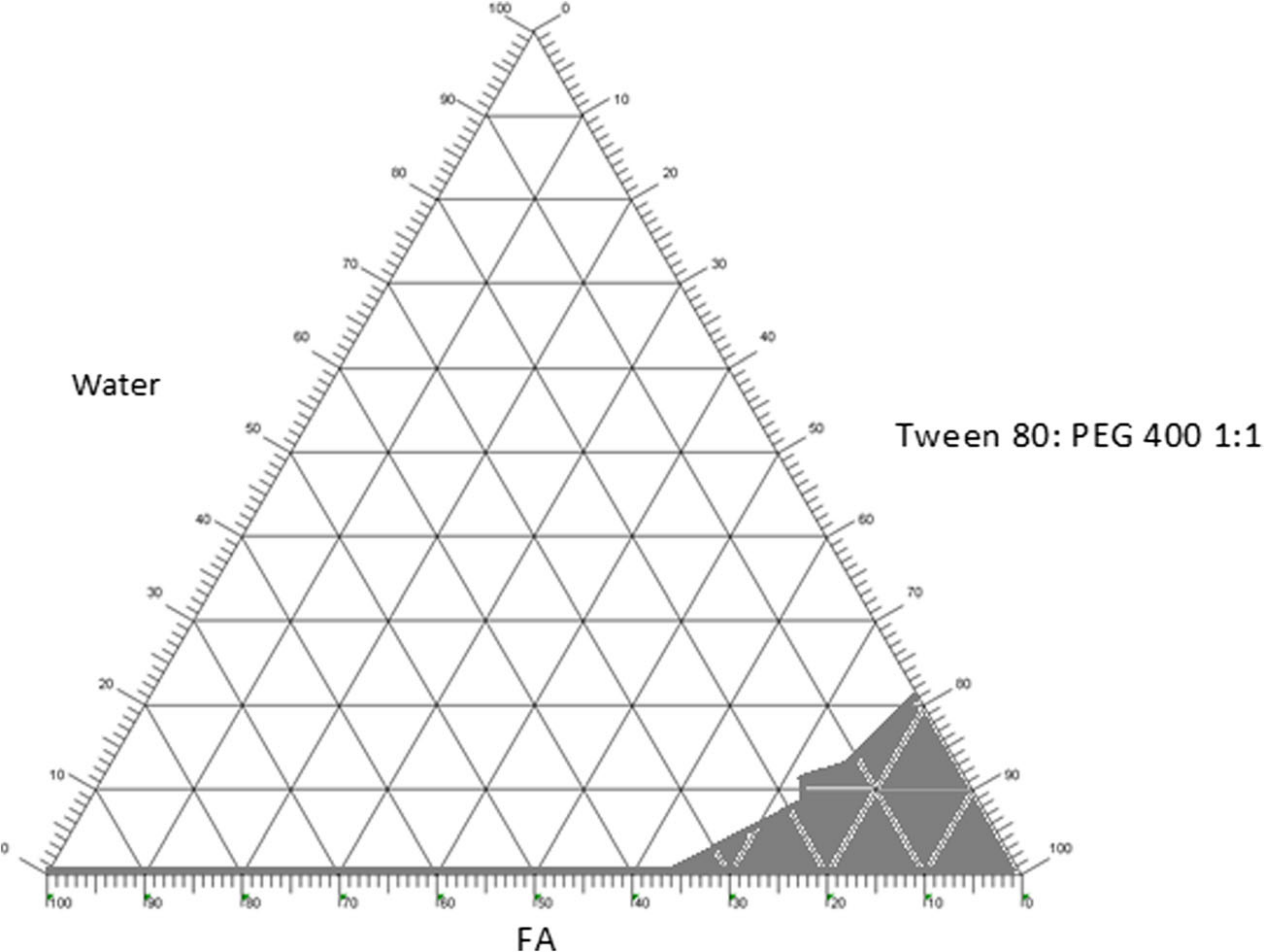
Pseudo-ternary phase diagram of α-linolenic acid, Tween 80 and PEG 400 (S/CoS mix 1:1) and water

According to the response surface analysis, Eqs. (1– 5) were generated and could be used to describe the effect of five selected variables on the peak area of fatty acids. The response surface analysis and the generated equations could be used to predict the peak area of the FAMEs and could be used to optimise the esterification conditions. Accordingly, the final optimum conditions that result in the most desirable value of the response (peak area of FAs) were found to be 0.5-ml volume of BCl_3_-methanol, 60 °C reaction temperature, 5-min reaction time and two extraction steps using 1 ml of n-hexane. These results indicated that the reaction temperature needs to be maximised to increase the peak area of all five FAs, whereas volume of catalyst, volume of n-hexane, reaction time and number of extraction steps needed to be kept low in order to achieve an optimal response. The significance of each parameter has been added and summarised in Supplementary Table 2.

1 $$ \mathrm{Y}1=3487-109.1\ \mathrm{X}1-811.9\ \mathrm{X}2+146.4\ \mathrm{X}3+98.38\ \mathrm{X}4-725.9\ \mathrm{X}5+34.38\ \mathrm{X}2*\mathrm{X}3+186.6\ \mathrm{X}2*\mathrm{X}5 $$

2 $$ \mathrm{Y}2=3382-175.6\ \mathrm{X}1-796.1\ \mathrm{X}2+94.63\ \mathrm{X}3+35.13\ \mathrm{X}4-609.1\ \mathrm{X}5-2.125\ \mathrm{X}2*\mathrm{X}3+185.6\ \mathrm{X}2*\mathrm{X}5 $$

3 $$ \mathrm{Y}3=3560-13.63\ \mathrm{X}1-806.9\ \mathrm{X}2-33.63\ \mathrm{X}3-27.12\ \mathrm{X}4-525.9\ \mathrm{X}5-56.62\ \mathrm{X}2*\mathrm{X}3-21.88\ \mathrm{X}2*\mathrm{X}5 $$

4 $$ \mathrm{Y}4=4442+119.1\ \mathrm{X}1-1019\ \mathrm{X}2+247.6\ \mathrm{X}3-535.4\ \mathrm{X}4-339.6\ \mathrm{X}5-448.9\ \mathrm{X}2*\mathrm{X}3-221.1\ \mathrm{X}2*\mathrm{X}5 $$

5 $$ \mathrm{Y}5=4756+137.7\ \mathrm{X}1-844.5\ \mathrm{X}2+24.75\ \mathrm{X}3-340.5\ \mathrm{X}4-933.8\ \mathrm{X}5-177.0\ \mathrm{X}2*\mathrm{X}3+134.0\ \mathrm{X}2*\mathrm{X}5 $$

### ATR and ^1^H NMR analysis

Formation of FAMEs was further confirmed by ATR and ^1^H NMR analyses. Both spectroscopic methods confirmed the changes in the structure of fatty acids after derivatisation and indicated the presence of esters in the samples without the formation of any artefacts. Formation of methyl esters can be confirmed by two characteristically strong absorption bands, methylene absorbance bands (wavenumber 1) and carbonyl stretching bands C=O (wavenumber 2) (Supplementary Figs. 1a–5a) [41].

ATR spectra of five fatty acids (C12:0, C13:0, C14:1, C16:1 and C18:3) and their methyl esters are shown in Supplementary Figs. 1a–5a. Fatty acids exhibited a strong O–H stretching band at 2850 cm^−1^ and a strong band at 1710 cm^−1^, due to the carbonyl bond C=O in free fatty acids. In methyl esters, methylene absorbance bands occurred at approximately 2920 cm^−1^, and carbonyl stretching bands C=O were shifted to approximately 1740 cm^−1^. This indicates the conversion of fatty acids into their methyl esters.

The ^1^H NMR spectra of the five fatty acids (C12:0, C13:0, C14:1, C16:1 and C18:3) and their methyl esters are shown in Supplementary Figs. 1b–5b. The characteristic intense peak of methyl ester protons was observed as a singlet at 3.622 ppm. This peak is the distinct peak for the confirmation of methyl esters. Other peaks appeared as triplet at 0.88 ppm caused by terminal methyl protons, a strong signal at 1.07–1.46 ppm related to methylene protons, a triplet and a multiplet at approximately 2.34 and 1.64 ppm, caused by two CH_2_ protons each, namely those α to the carboxyl group (C-2) and β to the carboxyl group (C-3), respectively. For C14:1 or C16:1 (unsaturated fatty acids), the signals of unsaturated protons appeared at approximately 5.40 ppm with integration value of two. A major peak was observed at about 2.00 ppm assigned to four allylic protons (at C4 and C7). In linolenic acid (C18:3) spectrum (Supplementary Fig. 5b), three double bonds (9c,12c,15c) did not cause any new peaks compared to myristoleic acid or palmitoleic acid, only changes in the integration values. The theoretical integration value of the olefinic protons increased to six while that of the large CH_2_ peak decreased further to 8. However, the linolenic acid spectrum showed two inner allylic methylene groups (CHCHCH_2_ CHCHCH_2_ CHCH) appearing at 2.8 ppm. Moreover, the peak of terminal methyl protons shifted downfield slightly, to about 0.98 ppm. Besides, BF_3_ in methanol has been reported to produce methoxy-substituted fatty acids as artefacts during the esterification of unsaturated fatty acids [26]. ATR and HNMR data showed that fatty acid derivatisation with BCl_3_-methanol was significantly efficient as no methoxy artefact with unsaturated fatty acids were identified.

### Method validation

#### Calibration curves and response linearity

As observed from the data (Table 3), the results were found to be linear over the concentration range of 25 to 200 μg ml^−1^ with coefficient of variations (*R* ^2^) greater than 0.9938 for all the fatty acids.

#### Limit of detection (LOD) and quantification (LOQ)

Limit of detection (LOD) and limit of quantitation (LOQ) were calculated from the slope of the linearity curve and standard deviation of line (also known as standard error of the predicted y-value for each x in the regression) by using Eqs. (6 and 7);

6 $$ LOD=3.3*\mathrm{R}/\mathrm{S} $$

7 $$ LOQ=10*\mathrm{R}/\mathrm{S} $$

where R is the standard deviation of the response and S is the slope of the calibration curve. As observed from Table 3, the limit of detection was within the range of 8 to 15 μg ml^−1^, and the limit of quantification was in the range of 24 to 46 μg ml^−1^ for the selected five fatty acids.

### Precision and accuracy

The precision and accuracy were evaluated through repeatability (intra-day) and reproducibility (inter-day) experiments. In order to check the precision and accuracy of the method, five standard concentrations were selected for each of the five FA (10, 50, 100, 150and 200 μg ml^−1^). The repeatability (intra-day) of the method was determined from six (*n* = 6) complete analyses of each sample under the same conditions in a day, and the reproducibility (inter-day) was determined from six (*n* = 6) complete analyses of each sample repeated three consecutive days. The values of the repeatability and reproducibility were expressed as relative standard deviation (RSD %). The accuracy of the method was evaluated through the recovery percentage (R %).

As shown in Table 3, values for intra-day RSD ranged between 2.49 and 5.11 % for all five fatty acids, and the values for inter-day RSD ranged between 3.02 and 4.71 %. The mean recovery percentage ranged from 97.45 to 100.38 % for all fatty acids.

The method validation results indicate that the developed method is linear and reproducible whereas variation between intra- and inter-day values is lower for all fatty acids. Moreover, recovery values are higher (almost approaching 100 %) for all fatty acids. Thus, these results indicate that the developed method is precise and accurate. Therefore, this method would be reliable to analyse a mixture of saturated and unsaturated fatty acids simultaneously.

The developed analytical method was used to quantify FA content in FA-based ME formulation.

### Development and characterisation of FA microemulsion

#### Construction of pseudo-ternary phase diagram

The pseudo-ternary phase diagram of the α-linolenic acid/Tween 80: PEG 400/water is shown in Fig. **4**. Ternary phase diagrams were constructed by taking 1:1 ratio of Tween 80 and PEG400. Tween 80 as non-ionic surfactant is widely applied in pharmaceutical preparations including ophthalmic preparations [42]. PEG 400 as hydrophilic co-solvents and/or co-surfactants is used to reduce the interface tension and increase the fluidity of the oil-water interface, thereby increasing the entropy of the system [43]. The shaded area of the phase diagram shows the ME region, whereas the non-shaded area displays the turbid region. Based on the phase diagram, three microemulsion formulations were selected from the ME region for anti-bacterial studies. The composition of the three microemulsion formulations is given in Table **4**.

**Table 4:** Composition and optimisation parameters of selected formulations

Formulation	Composition			Particle size ± SD (nm)	PDI ± SD	Zeta potential (mV)	Drug content mg/ml	Drug content %
	FA %	S/CoS %	Water%					
F1	4	91.5	4.5	350.50 ± 3.45	0.102 ± 0.012	0.0850 ± 0.016	38.88 ± 1.03	97.2
F2	3.5	78	18.5	281.90 ± 1.41	0.194 ± 0.023	−0.170 ± 0.022	33.95 ± 1.24	97
F3	16.5	79	4.5	315.80 ± 1.56	0.482 ± 0.052	−0.136 ± 0.054	161.95 ± 2.47	98.2

### Characterisation of selected microemulsion formulations

The results of droplet size, polydispersity index (PDI), zeta potential and drug content measurements are shown in Table **4**. The mean droplet size of the prepared microemulsions ranged between 281.9 ± 1.41 and 350.5 ± 3.45 nm, which was slightly higher than the usual microemulsion droplet size range of 20–200 nm. The higher droplet size could be due to the particle aggregation. The droplet size of F2 was less compared to F1 and F3. This could be due to the higher water concentration in F2 which lowers the viscosity and facilitate the formation of small particles. The PDI value for all formulations was less than 1 which is desirable with a lower PDI value signifying a higher uniformity of the droplet size in the formulation [44]. The drug content in the optimised formulations was measured using the developed GC method. The amount of the drug in the optimised formulations was around 97 and 98 % of the added amount which indicates that the developed GC method is precise and accurate to quantify the FA content in the ME formulation.

### Antibacterial activity of FAs and microemulsions against *S. aureus*

In this study, five fatty acids (LA, TA, MOA, POA, ALA) of various carboxyl lengths were investigated for their antimicrobial activity against *S. aureus*. After the initial screening, FAs were found with significant antimicrobial activity. The diameters of the zones of inhibition of the FAs are presented in Table 5. Overall, long-chain unsaturated FAs (MOA, POA, ALA) were more effective against *S. aureus* than the medium-chain saturated FAs (LA, TA). This trend is in agreement with the results of several other investigators who observed that unsaturated FAs have greater potency than saturated FAs [45, 46]. Amongst the long-chain unsaturated FAs, α-linolenic acid was selected for FA-based formulation because of its significant antimicrobial activity and cost-effectiveness.

**Table 5:** Means of inhibition growth diameter obtained by disc diffusion method using three selected FA-based ME formulations and individual components against *Staphylococcus aureus* mean ± SD% (*n* = 3)

Formulation	Zone of inhibition (mm)
F1	12.5 ± 0.7
F2	10.5 ± 0.7
F3	25.5 ± 0.7
FAs and active ingredients	
Tween 80	16.0 ± 1.4
PEG 400	8.5 ± 0.7
Lauric acid C12:0 at 1 mM	6.5 ± 0.2
Lauric acid C12:0 at 5 mM	10.2 ± 0.2
Tridecanoic acid C13:0 at 1 mM	7.2 ± 0.3
Tridecanoic acid C13:0 at 5 mM	13.5 ± 0.6
Myristoleic acid C14:1 at 1 mM	6.5 ± 0.5
Myristoleic acid C14:1 at 5 mM	10.2 ± 0.5
Palmitoleic acid C16:1 at 1 mM	9.5 ± 0.6
Palmitoleic acid C16:1 at 5 mM	11.5 ± 0.6
α-linolenic acid C18:3 at 1 mM	7.5 ± 0.6
α-linolenic acid C18:3 at 5 mM	12.3 ± 0.7

Before manufacturing FA formulations, an optimised analytical method was developed and validated to quantify fatty acids in the ophthalmic dosage form microemulsion.

The individual components (Linolenic acid, Tween 80 and PEG 400) and three formulated MEs were tested against *S. aureus* (Table 5). The results showed that *S. aureus* was susceptible to the three ME formulations and all individual components (Linolenic acid, PEG 400 and Tween 80). The results of the antimicrobial activity of PEG 400 are in agreement with the findings of Vaamonde et al. and Chirfe et al. [47] who found that concentrated PEG 400 solutions have significant antibacterial activity against various pathogenic bacteria, including *Klebsiella pneumoniae*, *Pseudomonas aeruginosa*, *Escherichia coli* and *S. aureus* [47]. F3 (16.5 % FA and 79 % S/CoS) that exhibited a high antibacterial zone compared to F1 (4 % FA and 91.5 % S/CoS) which showed medium antibacterial zone and F2 (3.5 % FA and 78 % S/CoS) which showed lowest antibacterial zone. Thus, F3 showed a strong inhibitory effect compared to F1 and F2. This could be due to the high concentration of Linolenic acid in F3. F2 showed a lowest inhibitory effect compared to F1 and F3, though there was a slight difference between F1 (4 %) and F2 (3.5 %) in terms of concentration of FA. So, this change in the antibacterial effect might be due to the high concentration of Tween 80 and PEG 400 in F1 (91.5 %) as compared to F2 (78 %). This could also be due to the high concentration of water in F2 (18.5 %) as compared to F1 and F3 (4.5 % water) which causes significant changes in the structure of ME that affects its antibacterial properties [48].

## Conclusion

The present study describes the formulation of FA-based microemulsions comprising of α-linolenic acid as oil phase, Tween 80 as surfactant, PEG 400 as co-surfactant and water as aqueous phase. The study developed a GC-FID method to detect five different FAs and to quantify the FA content in microemulsion (ME) formulations. Fatty acids were derivatised using BCl_3_-methanol as derivatisation reagent, and formation of FAMEs was confirmed by ATR and ^1^HNMR analyses. FAMEs were analysed by GC-FID with a high degree of accuracy, sensitivity and reliability. Moreover, the optimisation of the derivatisation method was carried out through a design of experiment (DoE) approach. The results indicated that the developed GC method is very effective for simultaneous detection of five FAs and to quantify the FA content in the microemulsion formulations. The antimicrobial efficacy of FA-based microemulsions was tested against *S. aureus*. It was concluded that the FA-based microemulsions have strong antimicrobial effect against *S. aureus*. These results clearly indicate that the developed FA-based microemulsions can be used for potential treatment of ophthalmia neonatorum.

## Electronic supplementary material

ESM 1 (DOCX 1135 kb)

## Abbreviations

FAs: Fatty acids
FAMEs: Fatty acid methyl esters
GC-FID: Gas chromatography-flame ionization detector
GC-MS: Gas chromatography-mass spectrometry
GLC: Gas liquid chromatography
HPLC: High-performance liquid chromatography
NMR: Nuclear magnetic resonance spectroscopy
ATR: Attentuated total reflectance
FTIR: Fourier transform infrared spectroscopy
LA: Lauric acid (C12:0)
TA: Tridecanoic acid (C13:0)
MOA: Myristoleic acid (C14:1)
POA: Palmitoleic acid (C16:1)
ALA: α-Linolenic acid (C18:3)
DoE: Design of experiment
BCl_3_: Boron trichloride
BF_3_: Boron trifluoride
