# Supplementary material for: Fatty acid microemulsion for the treatment of neonatal conjunctivitis: quantification, characterisation and evaluation of antimicrobial activity
Source: Drug Deliv Transl Res. 2016 Oct 20;6(6):722–34. doi: 10.1007/s13346-016-0338-3 (PMC5097082; doi:10.1007/s13346-016-0338-3)
Supplement: Supplementary file 1 — (DOCX 1135 kb) [file 13346_2016_338_MOESM1_ESM.docx]

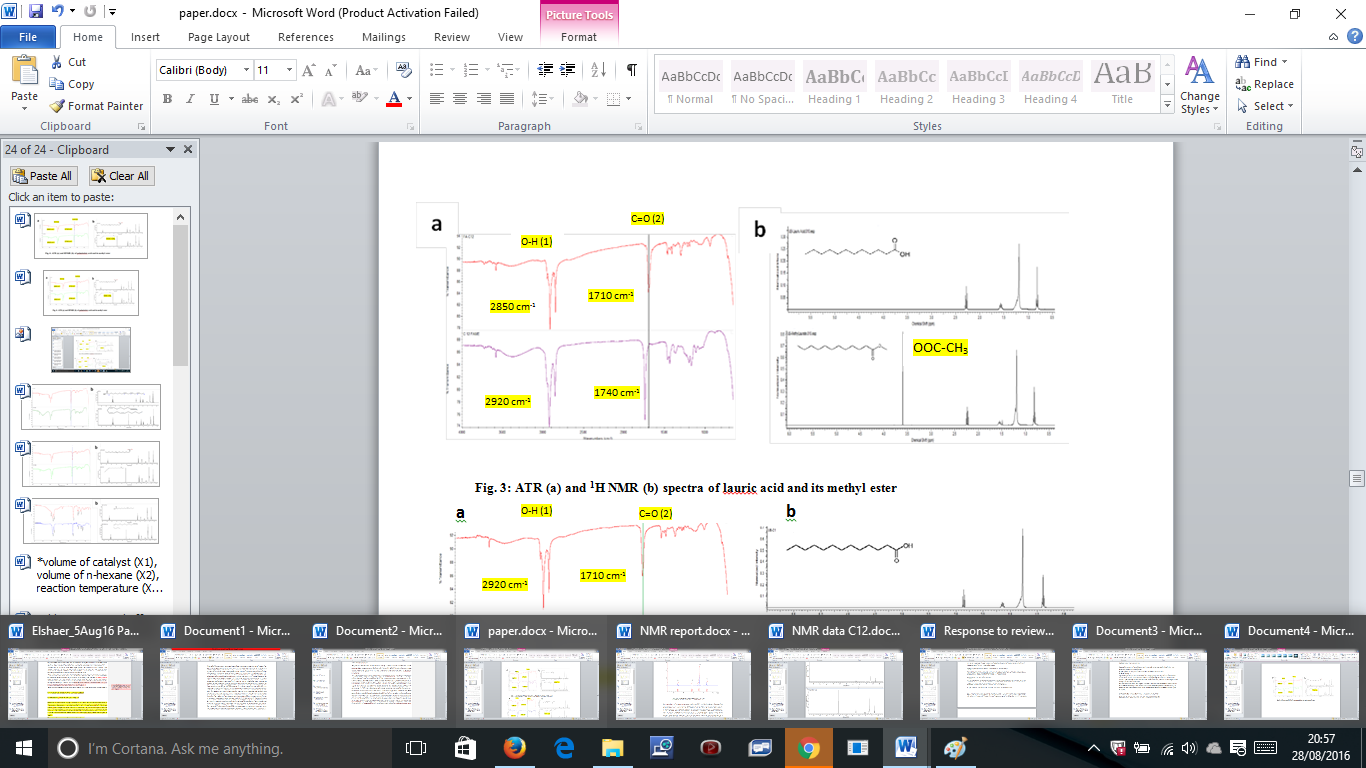
**Supplementary data:**


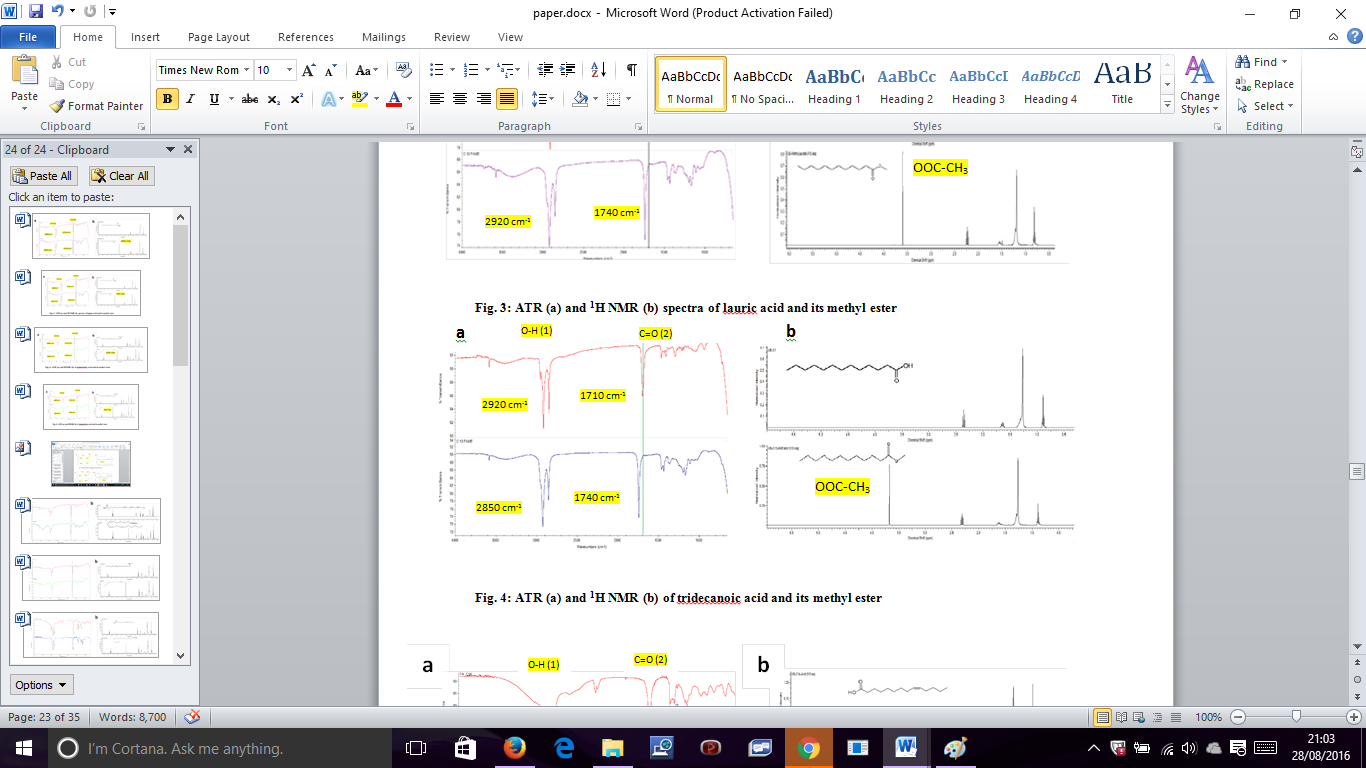
**Suppl. Fig. 1: ATR (a) and ^1^H NMR (b) spectra of lauric acid and its methyl ester**

**Suppl. Fig. 2: ATR (a) and ^1^H NMR (b) spectra of tridecanoic acid and its methyl ester**


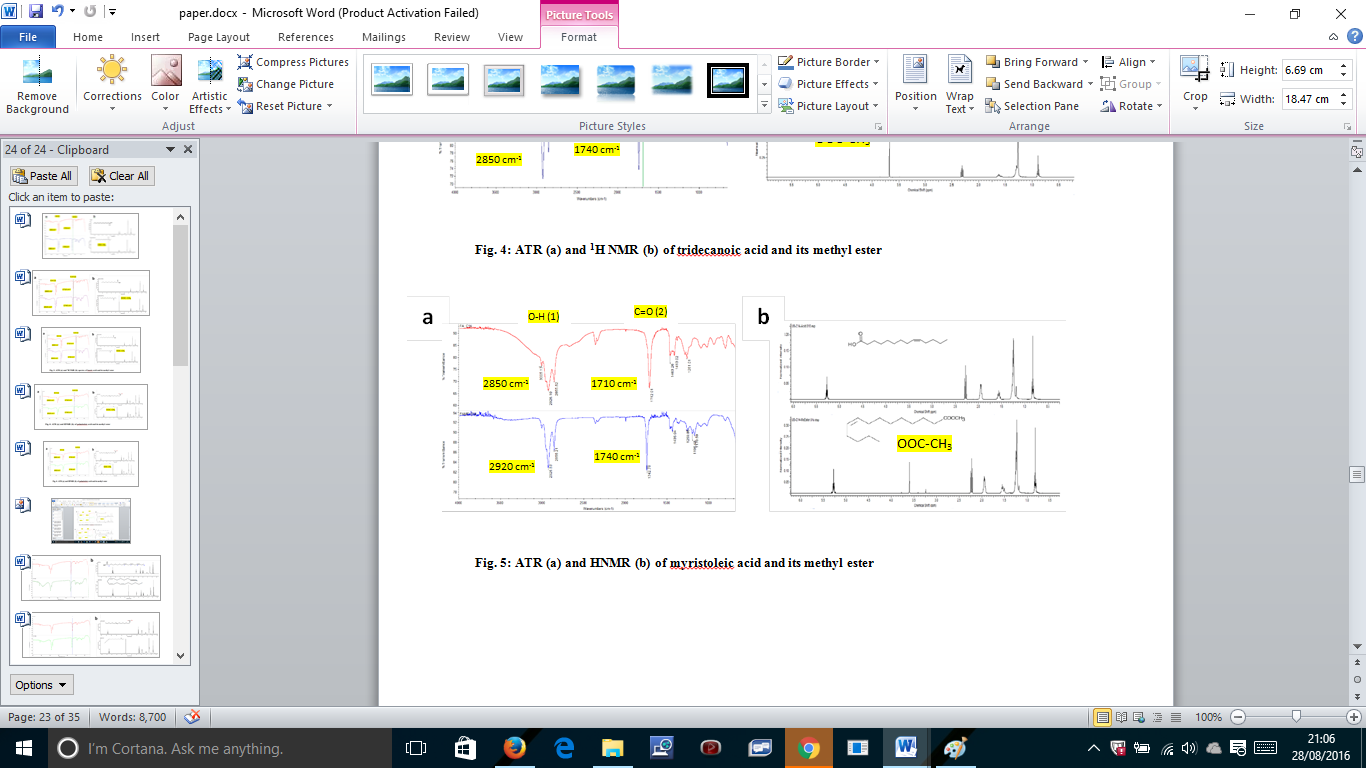


**Suppl. Fig. 3: ATR (a) and ^1^H NMR (b) spectra of myristoleic acid and its methyl ester**


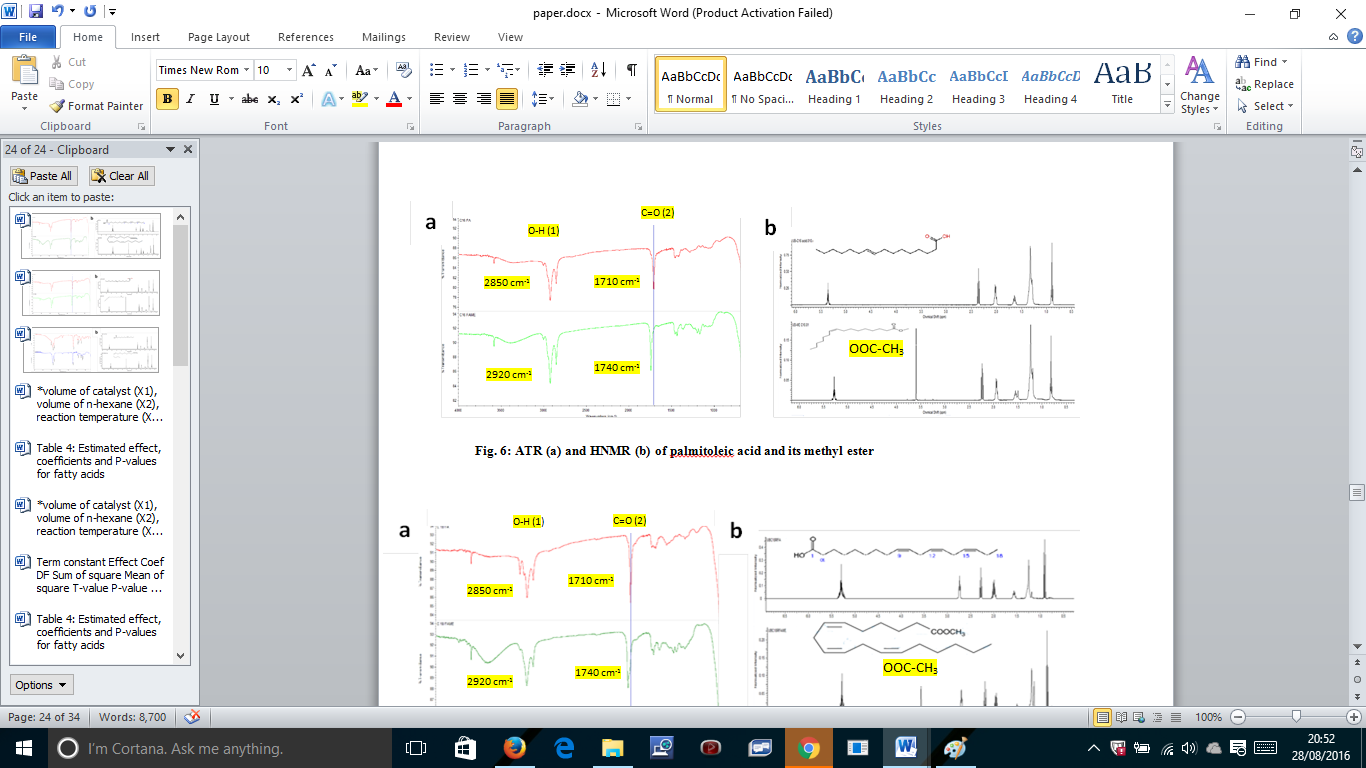


**Suppl. Fig. 4: ATR (a) and ^1^H NMR (b) spectra of palmitoleic acid and its methyl ester**


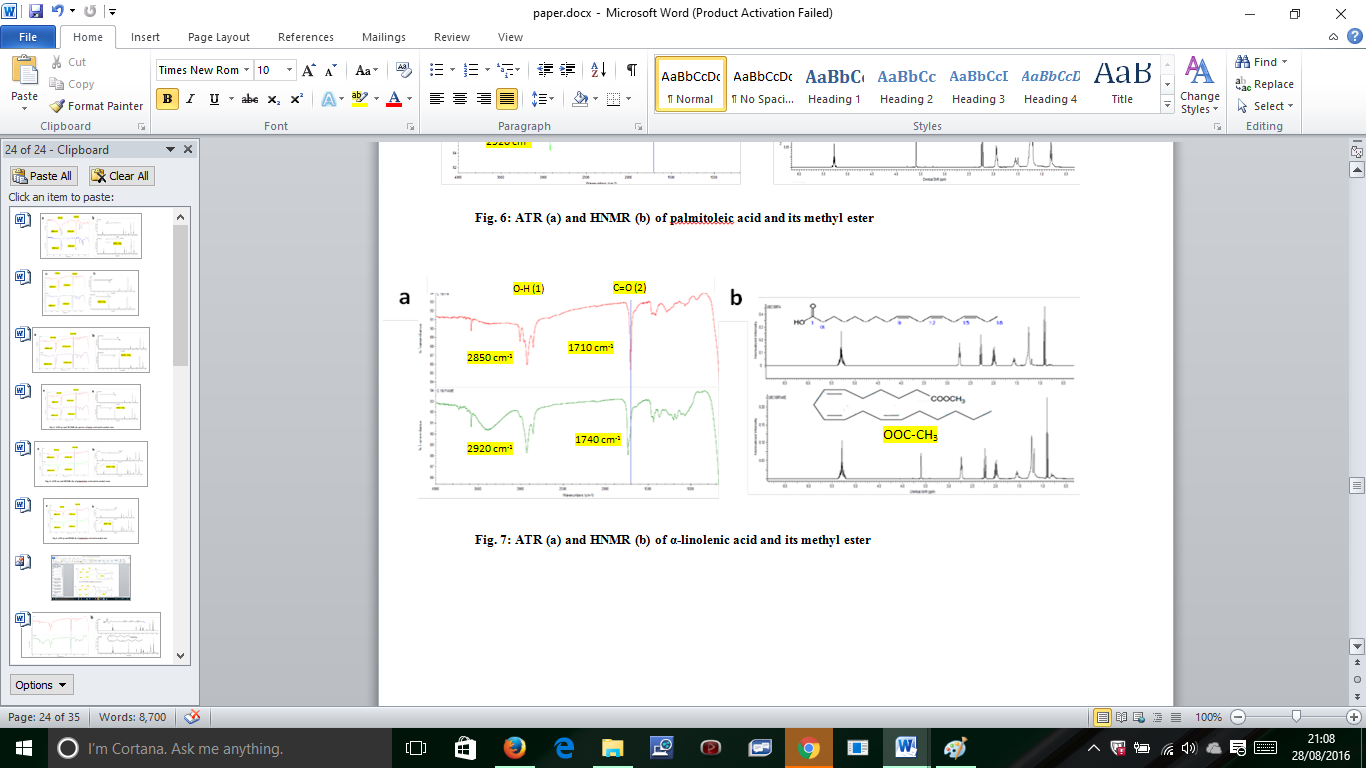


**Suppl. Fig. 5: ATR (a) and ^1^H NMR (b) spectra of α-linolenic acid and its methyl ester**

**Suppl. Table 1. Experimental results of peak areas of five fatty acids (**mean ± SD% (n = 3)).

| Sample number | Peak area C12:0 (Y_1_) | Peak area C13:0 (Y_2_) | Peak area C14:1 (Y_3_) | Peak area C16:1 (Y_4_) | Peak area C18:3 (Y_5_) |
| --- | --- | --- | --- | --- | --- |
| 1 | 2306±5.29 | 2114±4.35 | 2074±7.18 | 2245±2.20 | 2757±5.40 |
| 2 | 3291±3.40 | 3269±2.41 | 3899±2.87 | 6694±1.96 | 5213±1.61 |
| 3 | 4892±2.18 | 4665±1.90 | 4861±1.75 | 5538±2.02 | 6945±1.50 |
| 4 | 3023±3.40 | 2776±0.81 | 3350±0.64 | 3769±0.88 | 4661±2.64 |
| 5 | 5531±2.76 | 5280±1.42 | 4880±2.02 | 5622±1.57 | 6392±2.46 |
| 6 | 1966±6.25 | 2210±3.94 | 2336±4.92 | 3480±2.64 | 3467±0.56 |
| 7 | 3406±3.99 | 3242±1.54 | 3251±1.03 | 4199±2.52 | 4762±2.27 |
| 8 | 3482±3.30 | 3497±3.77 | 3826±2.48 | 3992±1.01 | 3853±3.11 |

**Suppl. Table 2: Estimated effect, coefficients and P-values for fatty acid**

| Term constant | **Effect** | **Coef** | **DF** | **Sum of square** | **Mean of square** | **T-value** | **P-value** |
| --- | --- | --- | --- | --- | --- | --- | --- |
| C12:0 (Y1) |  |  |  |  |  |  |  |
| X1 | -218.2 | -109.1 | 1 | 95266 | 95266 | -0.81 | 0.501 |
| X2 | -1623.8 | -811.9 | 1 | 5273128 | 5273128 | -6.05 | 0.026 |
| X3 | 292.7 | 146.4 | 1 | 171405 | 171405 | 1.09 | 0.389 |
| X4 | 196.7 | 98.4 | 1 | 77421 | 77421 | 0.73 | 0.540 |
| X5 | -1451.8 | -725.9 | 1 | 4215156 | 4215156 | -5.41 | 0.033 |
| C13:0 (Y2) |  |  |  |  |  |  |  |
| X1 | -351.2 | -175.6 | 1 | 246753 | 246753 | -1.34 | 0.313 |
| X2 | -1592.3 | -796.1 | 1 | 5070520 | 5070520 | -6.07 | 0.026 |
| X3 | 189.3 | 94.6 | 1 | 71631 | 71631 | 0.72 | 0.546 |
| X4 | 70.2 | 35.1 | 1 | 9870 | 9870 | 0.27 | 0.814 |
| X5 | -1218.3 | -609.1 | 1 | 2968266 | 2968266 | -4.64 | 0.043 |
| C14:1 (Y3) |  |  |  |  |  |  |  |
| X1 | -27.3 | -13.6 | 1 | 1485 | 1485 | -0.32 | 0.781 |
| X2 | -1613.8 | -806.9 | 1 | 5208378 | 5208378 | -18.80 | 0.003 |
| X3 | -67.3 | -33.6 | 1 | 9045 | 9045 | -0.78 | 0.515 |
| X4 | -54.3 | -27.1 | 1 | 5886 | 5886 | -0.63 | 0.592 |
| X5 | -1051.7 | -525.9 | 1 | 2212356 | 2212356 | -12.25 | 0.007 |
| C16:1 (Y4) |  |  |  |  |  |  |  |
| X1 | 238 | 119 | 1 | 113526 | 113526 | 0.34 | 0.768 |
| X2 | -2038 | -1019 | 1 | 8308926 | 8308926 | -2.88 | 0.102 |
| X3 | 495 | 248 | 1 | 490545 | 490545 | 0.70 | 0.556 |
| X4 | -1071 | -535 | 1 | 2293011 | 2293011 | -1.51 | 0.269 |
| X5 | -679 | -340 | 1 | 922761 | 922761 | -0.96 | 0.438 |
| C18:3 (Y5) |  |  |  |  |  |  |  |
| X1 | 275.5 | 137.7 | 1 | 151800 | 151800 | 0.88 | 0.473 |
| X2 | -1689.0 | -844.5 | 1 | 5705442 | 5705442 | -5.38 | 0.033 |
| X3 | 49.5 | 24.7 | 1 | 4901 | 4901 | 0.16 | 0.889 |
| X4 | -681.0 | -340.5 | 1 | 927522 | 927522 | -2.17 | 0.162 |
| X5 | -1867.5 | -933.8 | 1 | 6975112 | 6975112 | -5.95 | 0.027 |

*volume of catalyst (X_1_), volume of n-hexane (X_2_), reaction temperature (X_3_), reaction time (X_4_) and the number of extraction steps (X_5_)
